# Gγ subunit AT1/GS3-the “code” of alkaline tolerance in main graminaceous crops

**DOI:** 10.1007/s44154-023-00090-5

**Published:** 2023-04-25

**Authors:** Chuanfeng Ju, Cun Wang

**Affiliations:** 1 State Key Laboratory of Crop Stress Biology for Arid Areas, College of Life Sciences, Northwest Agriculture & Forestry University, Yangling, 712100 Shaanxi China; 2 Institute of Future Agriculture, Northwest Agriculture & Forestry University, Yangling, 712100 Shaanxi China

**Keywords:** Alkalinity tolerance, Gγ protein, Alkaline tolerance 1 (AT1), Aquaporins

## Abstract

This brief article highlights the results of Zhang et al. (Science 379, eade8416, 2023), who recently found that the Gγ subunit AT1/GS3 contributes to alkaline tolerance in several main monocots crops, and revealed the molecular mechanism of AT1/GS3-mediated response to alkaline stress in plants, which involves regulating H_2_O_2_ levels by inhibiting the phosphorylation of aquaporin PIP2s.

Salinized soil is divided into neutral pH saline soil (rich in NaCl and Na_2_SO_4_) and high pH saline-alkali soil (rich in NaHCO_3_ and Na_2_CO_3_, accounting for approximately 60%). According to the research of the Food and Agriculture Organization (FAO) of the United Nations in 2015 (https://www.fao.org/3/i5199e/i5199e.pdf), more than one billion hectares of salinized soil worldwide cannot be effectively utilized because of the high degree of salinization. Unreasonable fertilization and irrigation further aggravate the expansion of saline-alkali soil areas, posing a great threat to the sustainable development of agriculture.

Sorghum as the world's fifth largest crop has a strong resistance to alkalinity, drought, and barren soil. It is the main food source in arid and semi-arid regions of the world, and also the perfect model crop for mining saline-alkali tolerant gene resources (Ananda et al. 2020). Therefore, increasing the productivity of saline-alkali soils by cultivating alkalinity tolerant crops is an important approach to solve human food security and agricultural development in the future.

High alkalinity boosts the hydrogen peroxide (H_2_O_2_) levels and causes oxidative damage to cells, leading to apoptosis (An et al. 2020). Aquaporins (AQPs) can transport the redox signaling compound H_2_O_2_ to facilitate the fine regulation of H_2_O_2_ levels in the cytoplasm (Bienert and Chaumont 2014). However, how crops perceive alkaline stress and how the subsequent signal transduction affects H_2_O_2_ transport remain to be explored. G protein contains three subunits, Gα, Gβ, and Gγ, which play different roles in the signal perception, transduction, and downstream effector regulation (Pandey 2019). Although G protein signaling pathways have been well studied in mammals, the underlying mechanisms associated with the Gγ subunit in plant alkaline stress responses, remain unclear. This brief article highlights the recent results of a collaboration among Chinese scientists, who revealed an important alkalinity sensitivity gene, the Gγ subunit *AT1*/*GS3*, and manipulation of AT1/GS3 can significantly improve the alkaline tolerance of main crops and crop productivity in sodic lands, and clarified the molecular mechanism of AT1/GS3 involved in alkaline stress responses by inhibiting the phosphorylation of aquaporin PIP2s (Zhang et al. 2023).

The research group used the sorghum resource population to locate and clone a main effect site that was shown to be significantly related to sorghum alkaline tolerance through GWAS analysis, named AT1, which encodes a heterotrimeric G protein γ subunit (Gγ) homologous to the rice grain size regulation gene *OsGS3*, the maize gene *ZmGS3*, and the wheat gene *TaAT1* (Li et al. 2010; Mao et al. 2010; Wenjing et al. 2023). Haplotype analysis showed that there was a natural variation of frameshift mutation in *AT1* gene, which was significantly correlated with the variation in alkaline tolerance. To further investigate the function of AT1, the group constructed sorghum *AT1* overexpression lines, knockout lines, and near-isogenic lines (NILs). The alkaline stress phenotypes indicated that AT1 plays a negative role in the response of sorghum to alkaline stress. In addition, by analyzing the alkaline stress phenotypes of *AT1* ortholog gene-related materials in other crops, the group found that the role of AT1/GS3 in negatively regulating alkaline stress tolerance is highly conserved in other gramineous crops including rice, millet, and maize.

To reveal the molecular mechanism by which AT1 regulates alkaline stress responses in plants, Zhang et al. (2023) identified SbAT1-interacting proteins as the aquaporin proteins SbPIP2;1/2;2 and SbPIP1;3/1;4 by immunoprecipitation in combination with mass spectrometry (IP-MS), and confirmed their interactions by various protein interaction methods. Using the redox probe Cyto-roGFP2-Orp1 in combination with H_2_DCFDA (cytosolic redox status) and OxyBURST Green H_2_HFF BSA (apoplastic redox status) staining (Morgan et al. 2011; Oparka et al. 2016), they found that PIP2;1 promotes the efflux of H_2_O_2_ to reduce oxidative damage caused by alkaline stress, and AT1/GS3 inhibits the PIP2;1 phosphorylation levels to modulate the distribution of H_2_O_2_. Therefore, under alkaline stress conditions, the modification of *AT1* affects the phosphorylation of PIP2, thereby alleviating oxidative damage and endowing plants with high alkaline tolerance.

To further assess the usefulness of the modified *AT1*/*GS3* gene for crop yield in highly sodic lands containing natural alkali, they carried out field experiments in two sodic regions, the Daan region in Jilin Province and the Pinglou region in Ningxia Province. The results showed that the yield and biomass of rice, maize, sorghum, and millet improved by approximately 20–30% based on the alkaline tolerant allele AT1/GS3. In addition, the genetic modification of AT1 confers high alkaline tolerance to wheat (Wenjing et al. 2023). However, due to that *GS3* homolog plays opposite roles between dicots and monocots. For example, overexpressing *GS3* in rice resulted in reduced plant size and grain length, whereas overexpression of *GS3* in *Arabidopsis* increased flower organ and silique length, which may be due to *Arabidopsis* has not evolved related mechanism to be impacted by the tail-length difference in organ size regulation (Sun et al. 2018). Therefore, the genetic engineering of AT1/GS3 in dicotyledonous crops may be complicated and requires further investigation.

In summary, Xie Qi and collaborators have identified a key factor AT1/GS3 that negatively regulates alkaline tolerance in gramineous crops (including rice, maize, sorghum, millet, and wheat), and analyzed the molecular mechanism regulating the distribution of H_2_O_2_ by affecting the phosphorylation of aquaporin PIP2s, thereby maintaining ROS homeostasis and reducing the damage caused by alkaline stress in plants. Therefore, the gene editing of *AT1/GS3* can significantly improve the survival rate, yield, and biomass of crops in saline-alkali land (Fig. 1). At present, there are approximately 618 million hectares of saline-alkali land worldwide. If 20% of the medium and low saline-alkali land uses *AT1*/*GS3* gene resources, food production will increase by approximately 250 million tons per year.

**Fig. 1:**
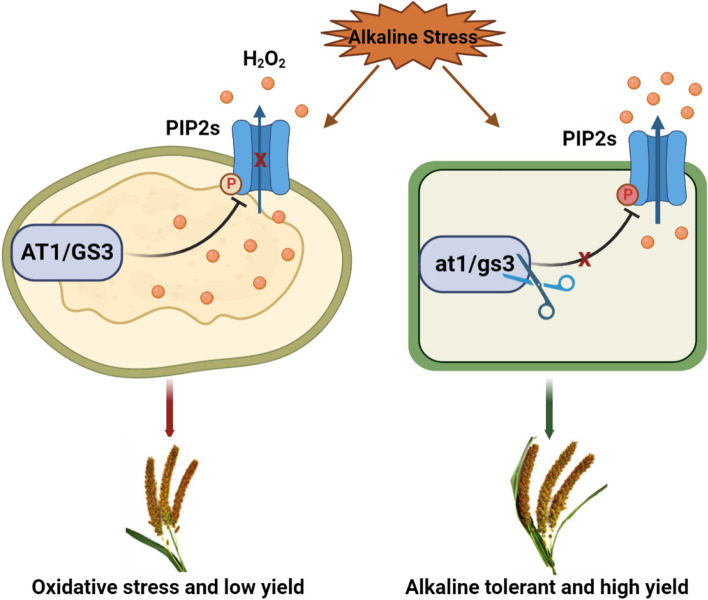
A proposed model of the Gγ subunit AT1/GS3-mediated alkaline stress responses in plants Oxidative stress occurs when plant cells are subjected to alkaline stress, and H2O2 produced which is toxic to cells, resulting in the failure of plants to complete the normal life cycle. The Gγ subunit AT1/GS3 inhibits the phosphorylation of aquaporin proteins PIP2s on the plasma membrane, resulting in excessive harmful H2O2 that cannot be pumped out of the cell timely and effectively, harming the growth and development of crops and resulting in low yield. Modification of AT1/GS3 can alleviate this toxicity, endow plants with high alkaline tolerance, and significantly increase yield

From basic research to technology and products, this study provides a guiding example for the application of genetic engineering in breeding genetic modification. By using elegant biochemical approaches, Xie and collaborators provide an effective way to alleviate alkaline damage and realize green agriculture, making a significant contribution to solving the global food security crisis and high-efficiency utilization of saline-alkali land. In addition, aquaporin PIP2s can not only transport H_2_O_2_, but more importantly facilitate the transport of water and small neutral solutes like carbon dioxide (CO_2_) and glycerin, which can enhance the drought endurance of plants. Therefore, whether and how AT1/GS3 affects the water transport ability of PIP2s and regulates drought sensitivity in crops requires further investigation. Furthermore, the salt sensor glycosyl inositol phosphorylceramide (GIPC) has been identified in the model plant *Arabidopsis thaliana* (Jiang et al. 2019). However, the specific situation regarding crops remains unclear. Since alkaline stress always co-occurs with saline stress, elucidating how plants perceive saline-alkali stress and the specific regulatory mechanisms, as well as the specificity and common effect between saline and alkaline stresses are still challenges and important directions for future research.

## Data Availability

Not applicable.
